# Perceived Prevalence, Awareness, and Attitude towards Counterfeit Medicines among Community Pharmacists of Kathmandu Valley: A Descriptive Cross-sectional Study

**DOI:** 10.31729/jnma.8651

**Published:** 2024-07-31

**Authors:** Sajala Kafle, Nisha Jha, Shital Bhandary, Pathiyil Ravi Shankar

**Affiliations:** 1 Department of Pharmacology, Patan Academy of Health Sciences, Lalitpur, Nepal; 2 Department of Clinical Pharmacology, KIST Medical College and Teaching Hospital, Lalitpur, Nepal; 3 Department of Community Health Sciences ana School of Public Health, Patan Academy of Health Sciences, Lalitpur, Nepal; 4 IMU Centre for Education, IMU University, Kuala Lumpur, Malaysia

**Keywords:** *Counterfeit drugs*, *community pharmacists*, *developing countries*, *cross-sectional studies*

## Abstract

**Introduction:**

Counterfeit medicines are a significant problem globally. In a developing country like Nepal, community pharmacists play an important role in dispensing medicines. The study was done to assess community pharmacists' perceived prevalence, awareness and attitude towards counterfeit medicines in Kathmandu valley.

**Methods:**

A descriptive cross-sectional study design was used. Data was collected conveniently from Kathmandu, Bhaktapur, and Lalitpur districts in March 2022 after obtaining ethical clearance from Nepal Health Research Council (reference no. 2200) and taking written informed consent from Community Pharmacists.

**Results:**

Among the 343 pharmacists who participated, 250 (72.89%) were from Kathmandu, 57 (16.62%) were from Lalitpur, and 36 (10.50%) from Bhaktapur. Most 252 (73.47%) were aged between 20 to 30 years, 222 (64.72%) were male, 239 (69.68%) had done a Diploma in Pharmacy, 201 (58.60%) had working experience of more than 5 years and 342 (99.71%) had done their education in Nepal. Their median perceived prevalence of counterfeit drugs was 10.00% but only 2.30% had clear knowledge of such practice. Out of 343, 332 (96.79%) believed that actions should be taken against community pharmacists dispensing counterfeit drugs, 325 (94.75%) believed that pharmacists who dispense counterfeit medicines are unprofessional; 338 (98.54%) stated that there should be strong law against counterfeit medicine sales.

**Conclusions:**

Median perceived prevalence of counterfeit medicines was 10.00% but only 2.30% were aware of counterfeit medicine dispensing. Out of 343 pharmacists, 332 (96.79%) mentioned that action should be taken against those pharmacists knowingly dispensing counterfeit medicines and 338 (98.54%) stated that there should be strong law against counterfeit medicines.

## INTRODUCTION

Counterfeit medicines are inferior in quality to the standard pharmaceutical products.^1^ They meet one of the following points: sample contaminated, drug containing less active pharmaceutical ingredient (API), drug containing no API, drug with less quantity of API.^2^ Counterfeit medicines (CM) are an important problem in lower- and middle-income countries.^3,4^ Drugs that are given by the oral route are more commonly available as counterfeit medicines (77%) compared to those given by the parenteral route (17%).^5^

In Nepal, people prefer to go to community pharmacists for treatment.^6,7^ Community pharmacists can help address problem of CM by dispensing safe, effective, and good-quality medicines to patients.^8,9^ A study revealed that 32.50% of the medicines in Nepal may be substandard.^10^ The aim of the study was to find out community pharmacists' perceived prevalence of as well as to assess awareness and attitude towards counterfeit medicines in all three districts of the Kathmandu valley.

## METHODS

A cross-sectional study was done among Community pharmacies (CPs) from Kathmandu Valley registered with the Department of Drug Administration after obtaining written informed consent.

The total number of allopathic community pharmacies registered with the DDA was 3881 (2828 in Kathmandu, 646 in Lalitpur, and 407 in Bhaktapur after removing pharmacies without names from the DDA list, which was used as the finite population of allopathic community pharmacies in this study).

The sample size was calculated using the formula:


$$\begin{array}{ll} \text{n} & ={\text{Z}}^{2}\times \frac{\text{p}\times \text{q}}{{\text{e}}^{\text{2}}}\\ & =1.96^{2}\times \frac{0.325\times 0.675}{0.05^{2}}\\ & =311 \end{array}$$

Where,

n = minimum required sample size
Z = 1.96 at 95% Confidence interval (CI)
p = prevalence of altered passive eruption (32.5%) taken from a previous study^10^
q = 1-p
e = margin of error, 5%

The calculated sample size was 311.

10% non response rate was added; n= n+ 10 % of n = 343

This sample size was distributed proportionally among the three districts with 250 (2828/3881*343) pharmacies for Kathmandu, 57 (646/3881*343) for Lalitpur, and 36 (407/3881*343) pharmacies for Bhaktapur district respectively. A convenience sampling method was used to collect the data from the Community Pharmacists working in the selected pharmacies as pretest study revealed difficulty to locate the randomly selected pharmacies in the field as per DDA list, which was a major limitation of this study in terms of external validity.

Participants working in community pharmacies in the Kathmandu valley registered with DDA and having required qualifications in pharmacy were included. They provided written, informed consent to participate.

The data were collected by trained data collectors using a semi-structured questionnaire. The names of the community pharmacies were de-identified while collecting data. A pre-validated questionnaire used in Lebanon^11^ to explore the problem of counterfeit medicines was used. The authors gave us the approval to use the questionnaire by email on 12^th^ August 2021. Pretesting of the questionnaire was done by administering it to 10 CPs from Lalitpur and Kathmandu. The participants who participated in the pretesting of the questionnaire were excluded from the main study. After the completion of the pretesting, the questionnaire was revised and finalized by the study team. The questionnaire was separated into three segments: the first segment collected information about the demographic features of the respondents, the second segment collected information about the professional responsibility of the community pharmacists, and the third segment collected information about counterfeit medicines. The data entry and data analysis were done using Statistical Package for the Social Sciences (SPSS) version 20 for windows. Descriptive statistics were used to summarize the results. Ethical approval was obtained from the Nepal Health Research Council with reference number 2200 dated 27^th^ February 2022.

## RESULTS

The minimum sample size after adjusting for The minimum sample size after adjusting for nonresponse was 343. In the present study 250 (72.89%) CPs were from Kathmandu; 255 (73.47%) were aged between 20 to 30 years, 222 (64.72%) were male, 239 (69.68%) had done Diploma in Pharmacy, 201 (58.60%) had working experience of more than 5 years and 342 (99.71%) had done their education in Nepal(Table 1).

**Table 1:** Demographic features of the study participants (n=343).

Variables	n (%)
**District**	
Kathmandu	250 (72.89)
Bhaktapur	36 (10.5)
Lalitpur	57 (16.62)
**Age**	
20-30 years	252 (73.47)
31-40 years	85 (24.78)
41-50 years	5 (1.46)
>51 years	1 (0.29)
**Gender**	
Male	222 (64.72)
Female	121 (35.28)
**Education level**	
Diploma	239 (69.68)
Bachelors	87 (25.36)
Masters	14 (4.08)
Others	3 (0.87)
**Working experience**	
<1 year	28 (8.16)
1-5 year	114 (33.24)
>5 years	201 (58.6)
**Country of education**	
Nepal	342 (99.71)
Abroad	1 (0.29)

The median perceived prevalence of counterfeit drugs was 10.00% with an inter-quartile range of 25.00%. The most reported perceived prevalence i.e. mode was 5.00%. Ironically, only 8 (2.33%) of the participants had knowledge of community pharmacists dispensing counterfeit drugs, 332 (96.79%) mentioned that action should be taken against CPs knowingly dispensing counterfeit drugs, 325 (94.75%) believed that pharmacists who dispense counterfeit drugs are unprofessional; and 338 (98.50%) stated that the law against counterfeit medicine sales should be strengthened. Further, 120 (34.98%) respondents mentioned that vitamins and supplements are most likely to be counterfeited. In addition to this, 176 (51.31%) strongly disagreed with the statement that pharmacists decide to stock counterfeit medicines since the quality is acceptable and 174 (50.73%) of the respondents mentioned that they have never been offered counterfeit medicines. Also, 339 (98.83%) of the respondents mentioned that they checked daily the integrity of the drug supplier and all of them mentioned that none of the products in their pharmacy have been confirmed as counterfeit products.(Table 2).

**Table 2:** Professional responsibility of pharmacists regarding counterfeit medicines (n= 343).

Statement	Strongly disagree	Disagree	Neutral	Strongly agree	Agree
Pharmacists who knowingly dispense counterfeit medicines are very clever	1 (0.29)	-	-	51 (14.87)	291 (84.84)
Pharmacists who knowingly dispense counterfeit medicines are good businessmen/women.	300 (87.46)	42 (12.24)	1 (0.29)		
Pharmacists who knowingly dispense counterfeit medicines are unprofessional	-	-	1 (0.29)	17 (4.96)	325 (94.75)
Pharmacists who knowingly dispense counterfeit medicines are unethical.	-	-	-	15 (4.37)	328 (95.63)
Pharmacists decide to stock counterfeit medicines in their pharmacy for easy money.			5 (1.46)	78 (22.74)	260 (75.8)
Pharmacists decide to stock counterfeit medicines in their pharmacy for the big profit.			1 (0.29)	77 (22.45)	265 (77.26)
Pharmacists decide to stock counterfeit medicines in their pharmacy since the quality is acceptable.	176 (51.31)	139 (40.52)	28 (8.16)	1 (0.29)	1 (0.29)
The law against counterfeit medicines should be strengthened.	1 (0.29)	-	-	-	342 (99.71)
The law against counterfeit salespeople should be strengthened.	1 (0.29)	-	-	4 (1.17)	338 (98.54)

Among the 343 respondents, 338 (98.54%) believed that counterfeit drugs can be identified by their cost and quality and 342 (99.71%) were not sure about whether they had purchased counterfeit medicines previously (Table 3).

**Table 3:** Questions about counterfeit products (n=343).

Question	Correct response n (%)
Other than medicine, are you willing to buy counterfeit products, given a good price and good quality?	
Yes	342(99.71)
No	1 (0.29)
Not	sure
Counterfeit products can be very dangerous.	
Yes	342(99.71)
No	1 (0.29)
Not	sure
Most counterfeit products are as good as the originals.	
Yes	1 (0.29)
No	337 (98.25)
Not sure	5(1.46)
Many branded original products are highly priced whereas counterfeit drugs are of better value.	
Yes	339 (98.83)
No	3 (0.87)
Not sure	1 (0.29)

## DISCUSSION

The current study was undertaken to find out the pharmacists awareness and attitude towards counterfeit medicines in Kathmandu Valley. This study found the perceived median prevalence of counterfeit drugs among community pharmacists of Kathmandu valley as 10.00% with interquartile range of 25.00% whereas a previous study found the actual measured prevalence of counterfeit medicines in the Kathmandu valley as 32.5%.^10^ This may be because in our study we measured only perceived prevalence with the help of following statement- "In your opinion what percentage of community pharmacists are aware of counterfeit medicines". However, in another study conducted in Kathmandu Valley laboratory analysis of medicines were done to measure the actual prevalence of counterfeit medicines.^10^

Most of the respondents 103 (30.03%) mentioned that only 5% of the medicines were counterfeit medicines. An assessment of the quality of substandard essential medicines in government healthcare facilities in Nepal found that 15.2% of medicines were substandard.^12^ This study used a questionnaire to study awareness of counterfeit medicines among CPs using a prevalidated questionnaire whereas the previous study examined registration compliance and laboratory tests were done to check the quality of the medicines.^10^ This study showed that CPs had good knowledge on certain aspects of counterfeit medicines while other aspects were low whereas another study published in 2015 showed that, there were presence of substandard medicines.^10^ This may be because of the difference in understanding between community pharmacists regarding different aspects of counterfeit medicines. However, another study did actual experiment on counterfeit medicines and calculated prevalence of 32.5%.^10^

In this study, only 8 (2.33%) reported that they were aware of CPs who dispensed counterfeit medicines. This observation is in contrast to another study conducted in Sudan where 86 % reported that they knew pharmacists who were dealing with counterfeit medicines.^13^ In another study, conducted in Iran, 36.90% of the participants stated that more than 50% of the community pharmacies supplied counterfeit medicines which were mentioned by 36.90% of participants.^14^ Also, 325 (94.75%) of the community pharmacists strongly agreed that the CPs dispensing counterfeit medicines were unprofessional and 328 (95.63%) strongly agreed that the community pharmacists dispensing counterfeit medicines are unethical. However, in another study conducted in Harar town, Ethiopia, 72.80% strongly agreed with the statement that prescribing and dispensing counterfeit drugs are unethical practices.^15^ Also in our study, 260 (75.80%) of the CPs believed that they are doing it for easy money and 265 (77.26%) strongly agreed that CPs deal with counterfeit medicines for a large profit. These findings were like another study conducted in Lebanon where 89.20 % of respondents mentioned that the CPs who dispense counterfeit medicines are unprofessional and 86.50% of the community pharmacists mentioned that dealing with counterfeit drugs is unethical. In addition to this, the respondents also mentioned that CPs who dispense counterfeit medicines do it for easy money as mentioned by 87.90 %, and large profit mentioned by 86.50 %.^11^

Our study showed that 339 (98.83%) of the CPs always checked daily for the integrity of the drug suppliers and depended on trusted drug distributors only. This data is similar to another study conducted in Egypt where 70.90% of the pharmacists always checked the honesty of the drug suppliers.^16^ Also, 176 (51.31%) of the community pharmacists strongly disagreed and 137 (39.94%) of the community pharmacists disagreed with the statement that the quality of counterfeit medicines is acceptably mentioned in the present study. However, in another study, it was mentioned by CPs that 66.80% of counterfeit medicines are inactive and 61.70% of the counterfeit medicines are harmful.^11^

The Drug Act 1978 of Nepal states that there is a provision for penalties for counterfeit medicines. It has been mentioned that if any medicine is not safe for public consumption or the quality of the medicine is compromised, then the manufacturer should get back all the medicines from sellers and distributors. Also, there is the provision of compensation. When the medicines manufactured are not safe or the quality of the drug has been compromised and it has caused the death of a person or injury of any kind then the drug manufacturer will be responsible for it. In this case, the drug manufacturer must provide compensation to the successor of the deceased of such death or to the person whose health has been compromised using the drug.^17^ However, implementing this law in a country like Nepal is a challenging task. In our study, 342 (99.71%) of the pharmacists mentioned that the law against counterfeit medicines should be strengthened which is higher compared to another study conducted in Sudan, where it was mentioned by 85% of the community pharmacists that there should be a strong law to address the problem of counterfeit medicines.^13^ Also, in our study, 120 (34.98%) of respondents mentioned that vitamins and supplements followed by nonregistered drugs at 109 (31.78%) were the common counterfeit medicines. This finding contrasts with the study conducted in Sudan, where antimalarial drugs were mentioned by 50% of the respondents to have a high risk for counterfeiting followed by antibiotics drugs mentioned by 16 (4.66%) of the respondents.^13^ Also in another study, drugs used in erectile dysfunction were the most likely drugs to be counterfeited which were mentioned by 74.90% of the pharmacists.^16^ Also, in a similar study conducted in Ethiopia, 45.70% of the community pharmacists mentioned that antibiotics were the most likely drugs to be counterfeited followed by analgesics which were mentioned by 34.70%.^15^

Furthermore, 116 (33.82%) of the pharmacists in this study mentioned that more than 90% of the community pharmacists in Kathmandu Valley are aware of counterfeit medicines. In addition, all the CPs mentioned that none of their products in the pharmacy has been confirmed as counterfeit drugs. However, in another study conducted in India, only 13.30% of respondents mentioned that they were able to identify counterfeit medicines from their labels.^18^

The most common age group of the respondents in our study was between 20 to 30 years and 222 (64.72%) of the participants in our study were male individuals. However, in a similar study conducted in Lebanon, it was stated that only 27.90 % of the participants were between the age group of 21 to 30 years and 57.50% were male individuals. ^11^ In addition to this, in our study, 201 (58.60%) of the CPs had prior work experience of more than 5 years. This finding is dissimilar to another study where 20.30% had prior experience of 5 to 10 years.^6^ Also, in our study 342 (99.71%) had done their studies in their home country, Nepal which is also distinct from another study where only 80.30% had conducted their study in their home country.^11^

The present study explored the pharmacist's perceived prevalence, awareness and views regarding counterfeit drugs only in the Kathmandu Valley and this can be regarded as the major weakness of the present study. The convenience sampling of community pharmacists is another limitation of this study for external validity. The findings show that there should be strong laws to manage the problem of counterfeit medicines by the government and the policymakers. Healthcare professionals, pharmacists, pharmaceutical companies as well as the public must be made aware of counterfeit medicines. Further research on counterfeit drugs can be done to explore awareness of counterfeit drugs among health professionals and the public. As we have looked at perception of community pharmacists regarding counterfeit medicines, there are no legal implications.

## CONCLUSIONS

Perceived median prevalence of counterfeit medicines was 10.00% but only 2.30% were aware of community pharmacists dispensing counterfeit medicines. Majority mentioned that action should be taken against those pharmacists knowingly dispensing counterfeit medicines and stated that there should be strong law against counterfeit medicines.
